# Partial genome sequence of the haloalkaliphilic soda lake bacterium *Thioalkalivibrio thiocyanoxidans* ARh 2^T^

**DOI:** 10.1186/s40793-015-0078-x

**Published:** 2015-10-26

**Authors:** Tom Berben, Dimitry Y. Sorokin, Natalia Ivanova, Amrita Pati, Nikos Kyrpides, Lynne A. Goodwin, Tanja Woyke, Gerard Muyzer

**Affiliations:** Microbial Systems Ecology, Institute of Biodiversity and Ecosystem Dynamics, University of Amsterdam, Amsterdam, The Netherlands; Winogradsky Institute of Microbiology, RAS, Moscow, Russia; Department of Biotechnology, Delft University of Technology, Delft, The Netherlands; Joint Genome Institute, Walnut Creek, CA USA

**Keywords:** Haloalkaliphilic, Soda lakes, Sulfur-oxidizing bacteria, Thiocyanate

## Abstract

*Thioalkalivibrio thiocyanoxidans* strain ARh 2^T^ is a sulfur-oxidizing bacterium isolated from haloalkaline soda lakes. It is a motile, Gram-negative member of the *Gammaproteobacteria*. Remarkable properties include the ability to grow on thiocyanate as the sole energy, sulfur and nitrogen source, and the capability of growth at salinities of up to 4.3 M total Na^+^. This draft genome sequence consists of 61 scaffolds comprising 2,765,337 bp, and contains 2616 protein-coding and 61 RNA-coding genes. This organism was sequenced as part of the Community Science Program of the DOE Joint Genome Institute.

## Introduction

Soda lakes are found in many arid zones across the world, such as the Kulunda Steppe in Russia, North-Eastern China, the Rift Valley in Africa, and in arid parts of North America, i.e. California and Nevada. The defining characteristics of these lakes are the abundance of carbonate/bicarbonate anions rather than chloride and their moderate to high salinities. This makes soda lakes a unique habitat with stable, alkaline pH values above nine and up to 11 [1]. Despite the high salinity and alkalinity, soda lakes harbor a rich microbial diversity that is responsible for highly active elemental cycles. Aside from the carbon cycle, the sulfur cycle is of great importance in these lakes [2], yet little is known about their precise biogeochemistry and dynamics [3]. A better understanding of these processes will lead to improved insights into the ecology and biogeochemical cycling in soda lakes. Additionally, sulfur-cycling extremophilic prokaryotes have important applications in bioremediation [4] and more detailed knowledge of their physiology may improve industrial waste processing. For these reasons, we have sequenced more than 70 strains belonging to the genus *Thioalkalivibrio*, a dominant cultivated group of chemolithoautotrophic haloalkaliphilic sulfur-oxidizing bacteria in soda lakes worldwide. Here we present the partial genome sequence of *Thioalkalivibrio thiocyanoxidans* ARh 2^T^.

## Organism information

### Classification and features

**Fig. 1:**
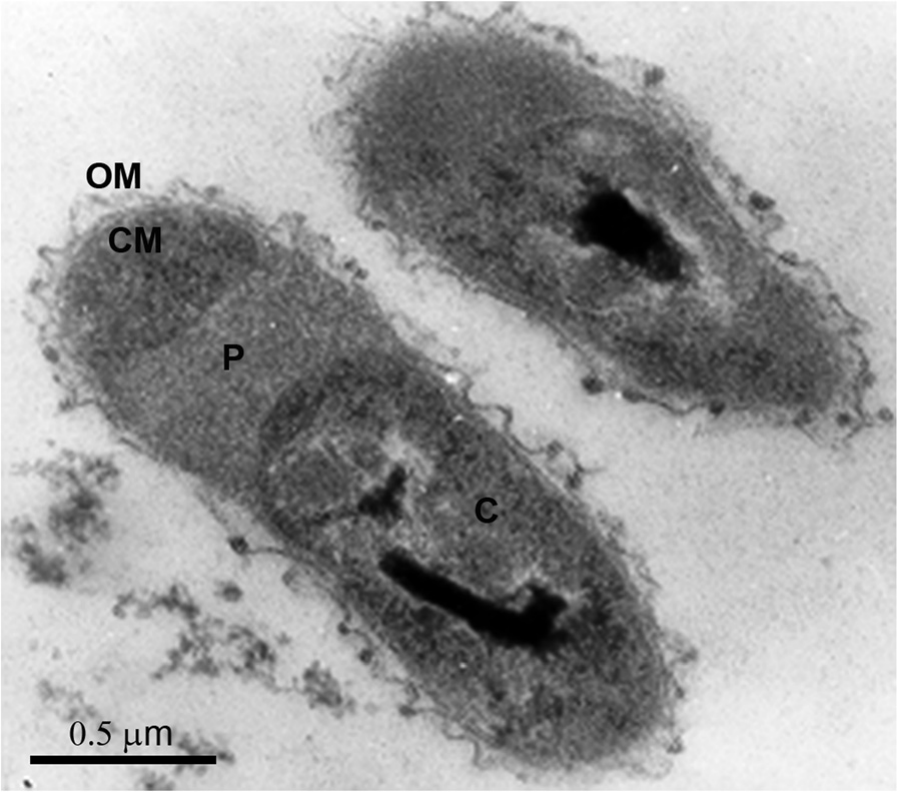
Thin section electron microscopy photograph of cells of strain ARh 2^T^ grown with thiocyanate in batch culture at pH 9.8 and 0.6 M total Na^+^. OM - outer cell membrane; CM - cytoplasmic membrane; P - periplasm; C - cytoplasm

**Fig. 2:**
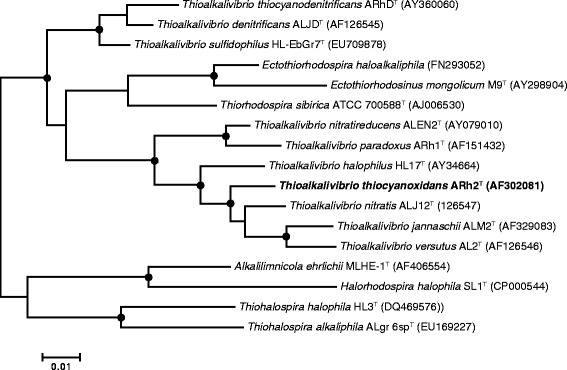
Phylogenetic tree based on 16S rRNA sequences comprising the *Thioalkalivibrio* type strains and several other members of the *Ectothiorhodospiraceae* family. Black dots mark nodes with a bootstrap value between 90 and 100 %. 16S rRNA sequences of members of the *Alphaproteobacteria* were used as the outgroup, but pruned from the tree. The tree was constructed using ARB [21] and bootstrap values calculated using MEGA6 [22]

*T. thiocyanoxidans* ARh 2^T^ forms motile vibrio-like cells of approximately 0.5–0.6 by 0.8–1.4 μm (basic properties are summarized in Table 1). The cells grown with thiocyanate as electron source have a remarkably extended periplasm (Fig. 1). It is a Gram-negative bacterium belonging to the *Gammaproteobacteria* (Fig. 2). The species description is based on four strains (ARh 2, ARh 3, ARh 4 and ARh 5) that were isolated from sediment samples of South-Western Siberian, Kenyan and Egyptian soda lakes. Strain ARh 2 is a type strain of the *T. thiocyanoxidans* species. As a chemolithoautotroph, ARh 2^T^ derives energy from the oxidation of inorganic sulfur compounds, such as sulfide, thiosulfate, thiocyanate, elemental sulfur and polysulfides. The most interesting properties are its ability to grow on thiocyanate as the sole source of energy, sulfur and nitrogen and its ability to grow in saturated soda brines brines with thiosulfate as energy source [5].

**Table 1:** Classification and general features of *Thioalkalivibrio thiocyanoxidans* ARh 2^T^ [12]

MIGS ID	Property	Term	Evidence code^a^
	Classification	Domain *Bacteria*	TAS [13]
		Phylum *Proteobacteria*	TAS [14, 15]
		Class *Gammaproteobacteria*	TAS [15, 16]
		Order *Chromatiales*	TAS [15, 17]
		Family *Ectothiorhodospiraceae*	TAS [18]
		Genus *Thioalkalivibrio*	TAS [19]
		Species *Thioalkalivibrio thiocyanoxidans*	TAS [5]
		Type strain: ARh 2^T^ (DSM 13532)	
	Gram stain	Negative	TAS [5, 19]
	Cell shape	Vibrios	TAS [5]
	Motility	Motile	TAS [5]
	Sporulation	Non-sporulating	NAS
	Temperature range	Mesophilic	TAS [5]
	Optimum temperature	35–37 °C	TAS [5]
	pH range; Optimum	8.5–10.5	TAS [5]
	Carbon source	Inorganic carbon	TAS [5]
MIGS-6	Habitat	Soda lakes	TAS [5]
MIGS-6.3	Salinity	0.3–4.3 M Na^+^	TAS [5]
MIGS-22	Oxygen requirement	Aerobe	TAS [5]
MIGS-15	Biotic relationship	Free-living	NAS
MIGS-14	Pathogenicity	Non-pathogenic	NAS
MIGS-4	Geographic location	Kenya	TAS [5]
MIGS-5	Sample collection	1999	TAS [5]
MIGS-4.1	Latitude	Not reported	
MIGS-4.2	Longitude	Not reported	
MIGS-4.4	Altitude	Not reported	

^a^Evidence codes - IDA: Inferred from Direct Assay; TAS: Traceable Author Statement (i.e., a direct report exists in the literature); NAS: Non-traceable Author Statement (i.e., not directly observed for the living, isolated sample, but based on a generally accepted property for the species, or anecdotal evidence). These evidence codes are from the Gene Ontology project [20]

## Genome sequencing information

### Genome project history

*Thioalkalivibrio thiocyanoxidans* ARh 2^T^ was sequenced as part of a project aimed at sequencing a large number of *Thioalkalivibrio* isolates. The goal of this project is to enable the study of the genomic diversity of the dominant genus of sulfur-oxidizing bacteria in soda lakes. *T. thiocyanoxidans* ARh 2^T^ was selected for its ability to grow in salt-saturated brines (4.3 M Na^+^) and for its ability to grow on thiocyanate as the sole energy, sulfur and nitrogen source. The permanent draft genome we present here consists of approximately 2.8 million basepairs divided over 61 scaffolds. Sequencing was performed at the Joint Genome Institute under project 1008667. The genome sequence was released in Genbank on December 25, 2014. An overview of the project is given in Table 2.

**Table 2:** Project information

MIGS ID	Property	Term
MIGS 31	Finishing quality	Improved high-quality draft
MIGS-28	Libraries used	Illumina standard fragment, 270 bp
MIGS 29	Sequencing platforms	Illumina HiSeq 2000
MIGS 31.2	Fold coverage	1819
MIGS 30	Assemblers	Velvet 1.1.04 [7], ALLPATHS R39750 [8]
MIGS 32	Gene calling method	Prodigal [9], GenePRIMP [10]
	Locus Tag	G372
	Genbank ID	ARQK00000000
	GenBank Date of Release	2014-12-25
	GOLD ID	Gp0025980
	BIOPROJECT	PRJNA185302
	IMG submission ID	12214
MIGS 13	Source Material Identifier	DSM 13532
	Project relevance	Biotechnology

### Growth conditions and genomic DNA extraction

*T. thiocyanoxidans* ARh 2^T^ (DSM 13532) was cultured in a standard buffer containing sodium carbonate and bicarbonate at pH 10. The total salt concentration was 0.6 M Na^+^ [6]. The energy source was thiosulfate, at a concentration of 40 mM. After harvesting, the cells were stored at −80 °C for further processing. Genomic DNA was extracted using a chloroform-phenol-isoamylalcohol mixture and precipitated with ethanol. After vacuum drying, the pellet was dissolved in water and the quantity and quality of the DNA determined using the JGI-provided Mass Standard Kit.

### Genome sequencing and assembly

This strain was sequenced as part of the Community Science Program of the US Department of Energy Joint Genome Institute. The Illumina HiSeq 2000 platform was used for sequencing, with a depth of 1819X. More details regarding the library construction and sequencing are available at the JGI website. Reads were filtered using DUK and assembled using Velvet 1.1.04 [7]. Pseudoreads (1–3 Kb) were generated from the Velvet output using wgsim and reassembled using ALLPATHS-LG r42328 [8]. The final assembly consists of 61 scaffolds.

### Genome annotation

Genes were predicted using Prodigal [9], followed by a round of manual curation using GenePRIMP [10] to detect pseudogenes. The resulting predicted genes were translated and annotated using the NCBI NR database in combination with the UniProt, TIGRFam, Pfam, KEGG, COG and InterPro databases and tRNAScanSE [11] for tRNA prediction. Ribosomal RNAs were detected using models built from SILVA. Further annotation was performed using the Integrated Microbial Genomes platform. All annotation data is freely available there, with IMG submission ID 12214.

## Genome properties

The final draft of the genome comprises 2.8 million base pairs in 61 scaffolds, with a G + C percentage of 66.18 %. The gene calling and annotation pipeline detected 2677 genes, of which 2616 code for proteins. Basic statistics concerning the genome sequence are shown in Table 3. In total, 70 % of the genes could be assigned functional categories based on COGs (see Table 4).

**Table 3:** Genome statistics

Attribute	Value	% of Total
Genome size (bp)	2,765,337	100.00
DNA coding (bp)	2,496,809	90.29
DNA G + C (bp)	1,829,984	66.18
DNA scaffolds	61	100.00
Total genes	2677	100.00
Protein coding genes	2616	97.72
RNA genes	61	2.28
Pseudo genes	Not determined	Not determined
Genes in internal clusters	Not determined	Not determined
Genes with function prediction	2230	83.30
Genes assigned to COGs	1885	70.41
Genes with Pfam domains	1799	78.94
Genes with signal peptides	217	8.11
Genes with transmembrane helices	655	24.47
CRISPR repeats	1	100.00

**Table 4:** Number of genes associated with the 25 general COG functional categories

Code	Value	% age	Description
J	148	7.09	Translation, ribosomal structure and biogenesis
A	1	0.05	RNA processing and modification
K	70	3.36	Transcription
L	98	4.70	Replication, recombination and repair
B	2	0.10	Chromatin structure and dynamics
D	32	1.53	Cell cycle control, Cell division, chromosome partitioning
V	29	1.39	Defense mechanisms
T	105	5.03	Signal transduction mechanisms
M	153	7.33	Cell wall/membrane biogenesis
N	73	3.50	Cell motility
U	72	3.45	Intracellular trafficking and secretion
O	109	5.23	Posttranslational modification, protein turnover, chaperones
C	148	7.09	Energy production and conversion
G	82	3.93	Carbohydrate transport and metabolism
E	145	6.95	Amino acid transport and metabolism
F	60	2.88	Nucleotide transport and metabolism
H	131	6.28	Coenzyme transport and metabolism
I	86	3.02	Lipid transport and metabolism
P	105	5.03	Inorganic ion transport and metabolism
Q	37	1.77	Secondary metabolites biosynthesis, transport and catabolism
R	228	10.93	General function prediction only
S	195	9.35	Function unknown
-	792	29.59	Not in COGs

The total is based on the total number of protein coding genes in the genome

## Conclusions

Sequencing of the genome of *Thioalkalivibrio thiocyanoxidans* ARh 2^T^ is an important step towards a more comprehensive understanding of the mechanism by which this organism can adapt to extremely high salinity. In addition, it will provide important information on the role of this organism in the carbon and sulfur cycles of natural and engineered environments, in particular in the degradation of thiocyanate.
